# *Parachlamydiaceae*: Potential Emerging Pathogens

**DOI:** 10.3201/eid0806.010210

**Published:** 2002-06

**Authors:** Gilbert Greub, Didier Raoult

**Affiliations:** * Unité des Rickettsies, Université de la Méditerranée, Marseille, France

**Keywords:** *Parachlamydiaceae*, pathogenicity, free-living amoebae

## Abstract

*Parachlamydiaceae*, which naturally infect amoebae, form a sister taxon to the *Chlamydiaceae* on the basis of the *Chlamydia*-like cycle of replication and 80% to 90% homology of ribosomal RNA genes. Because intra-amoebal growth could increase the virulence of some intracellular bacteria, *Parachlamydiaceae* may be pathogenic. Arguments supporting a pathogenic role are that *Chlamydia pneumoniae*, a well-recognized agent of pneumonia, was shown to infect free-living amoebae and that another member of the *Chlamydiales*, *Simkania negevensis*, which has 88% homology with *Parachlamydia acanthamoebae,* has caused pneumonia in adults and acute bronchiolitis in infants. The recent identification of a 16S rRNA gene sequence of a *Parachlamydiaceae* from bronchoalveolar lavage is additional evidence supporting potential for pathogenicity.

Nosocomial pneumonia, a frequent complication associated with considerable illness and death (*1*,*2*), is the leading cause of death from nosocomial infections (*3*). Community-acquired pneumonia, which is also common, is associated with a case-fatality rate of up to 8.8% (*4*). Despite use of standard diagnostic methods, no microbial cause could be identified in 47% to 55% of community-acquired pneumonia worldwide in adults (*5*–*7*) and 20% to 75% of nosocomial pneumonia (*8*,*9*). Emerging intracellular bacteria, which grow poorly or not at all on media used routinely for detecting human pathogens from clinical samples, could be the causative agents of these pneumonias of unknown etiology. During recent decades, several previously unrecognized intracellular bacteria have been discovered through the genotypic approach. In addition, use of amoebal coculture procedures (*10*) allows recovery of some fastidious gram-negative bacteria, such as the *Legionella*-like amoebal pathogens (*11*,*12*), *Candidatus* *Odyssella thessalonicensis* (*13*), *Sacrobium lyticum* (*14*), several *Afipia* species (*15*), and *Chlamydia*-like endosymbionts (*16*,*17*).

## Amoebae: Microbial Trojan Horses

Although *Legionella* was the first pathogen demonstrated to multiply and persist in amoebae (*18*), several other fastidious intracellular bacterial pathogens, including *Chlamydia pneumoniae* (*19*), *Mycobacterium avium* (*20*), *Listeria monocytogenes* (*21*), and an *Ehrlichia*-like organism (*22*), may infect free-living amoebae. Extensive study of the ecology of *Legionella pneumophila* has confirmed empirical observations of its predilection for growth in hot water tanks and its localization in sediment (*23*). Rowbotham described the ability of *L. pneumophila* to multiply intracellularly within protozoa (*18*) and suggested that free-living amoebae could be a reservoir for *Legionella* species (*24*). As amoebae are common inhabitants of natural aquatic environments and water systems (*25*,*26*) and are resistant to extreme temperatures, pH, and osmolarity conditions while encysted (*27*), the *Legionella* reservoir is important. Growth of free-living amoebae at high temperatures (44°C to 53°C) was observed more frequently for strains isolated from hot-water tanks (mainly *Hartmanella vermiformis*) than for those isolated from moist sanitary areas (mainly *Acanthamoeba*, *Naegleria,* and *Valkhampfia* species) (*26*). This great tolerance of cysts and species-dependent thermotolerance of trophozoites could account for the difficulty in eliminating Legionellae from water systems (*28*). The resistance of *Acanthamoeba* spp. cysts to various disinfecting solutions (*29*–*31*) complicates the eradication of free-living amoebae. Moreover, a wide variety of *Enterobacteriaceae* have increased resistance to chlorination when ingested by *Tetrahymena pyriformis* (*32*). Thus, free-living amoebae could readily act as Trojan horses for bacterial endosymbionts (*33*,*34*).

The relationship between *Legionellaceae* and free-living amoebae, which serves as a model for other endosymbionts such as *Parachlamydiaceae*, is not restricted to the role of reservoir. Indeed, *Acanthamoeba* strains were found to produce *Legionella*-containing vesicles, which may be agents of transmission of legionellosis. The risk of transmission may be underestimated by plate count methods (*35*). In addition, Legionellae grown inside amoebae were more virulent (*36*,*37*), more motile (*24*), and more resistant to biocides (*38*) than are bacteria cultured in axenic media. The entry of Legionellae into monocytes was found to be enhanced by the intra-amoebal growth environment (*39*). In addition, intra-amoebal growth of *L. pneumophila* was shown to induce an antibiotic-resistant phenotype, while Legionellae cultured in broth did not (*40*). Similarly, *M. avium* living within *Acanthamoeba* had greater resistance to rifabutin, clarithromycin, and azithromycin than did strains living in macrophages (*41*). This finding could result from decreased uptake of antibiotics into the amoebae, an inactivation of the compound within amoebae, or a change in the bacterial phenotype. Replication of bacteria in amoebae was found not only to affect the bacterial host (through increased potential for spread, resistance to biocides and antibiotics, and acquisition of virulence traits) but also to enhance the pathogenicity of the free-living amoebae (*42*).

## The *Parachlamydiaceae*

**Figure 1:**
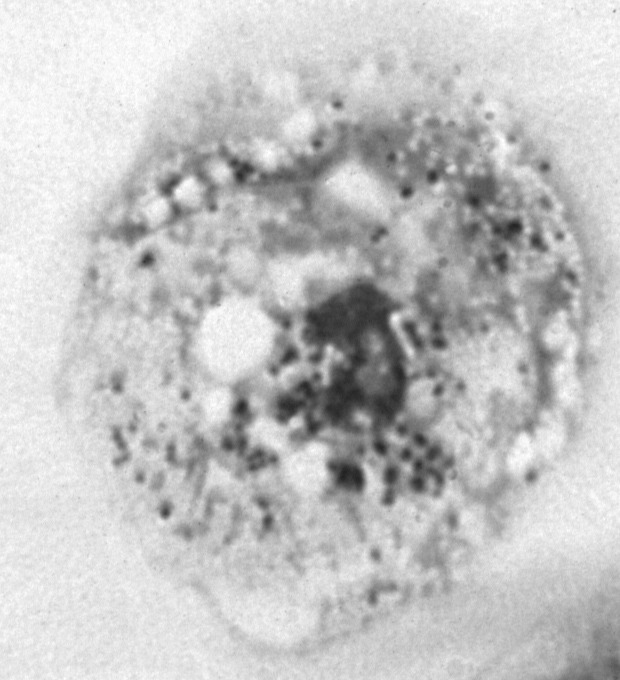
Hall’s coccus within *Acanthamoeba polyphaga.* Diff Quick staining (Dade, Boehring, Paris, France). Magnification X 1,000.

**Figure 2:**
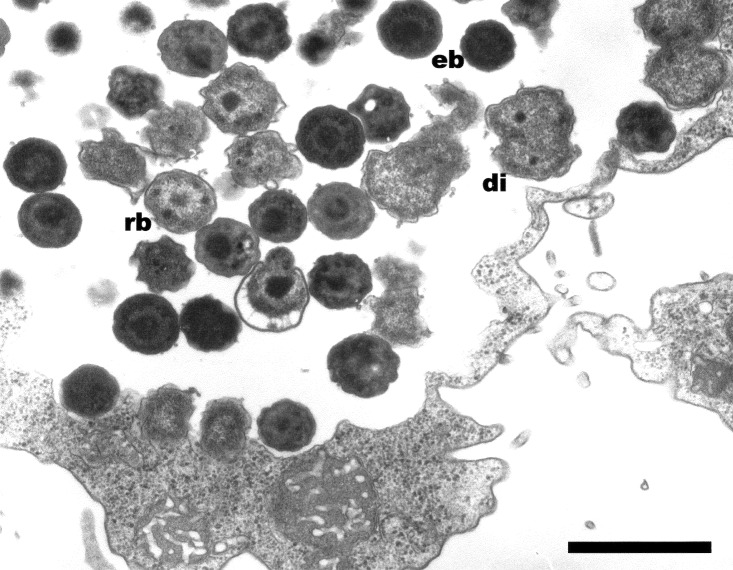
Hall’s coccus within *Acanthamoeba polyphaga.* Electron microscopy, magnification X 12,000, bar = 1 µm.

**Figure 3:**
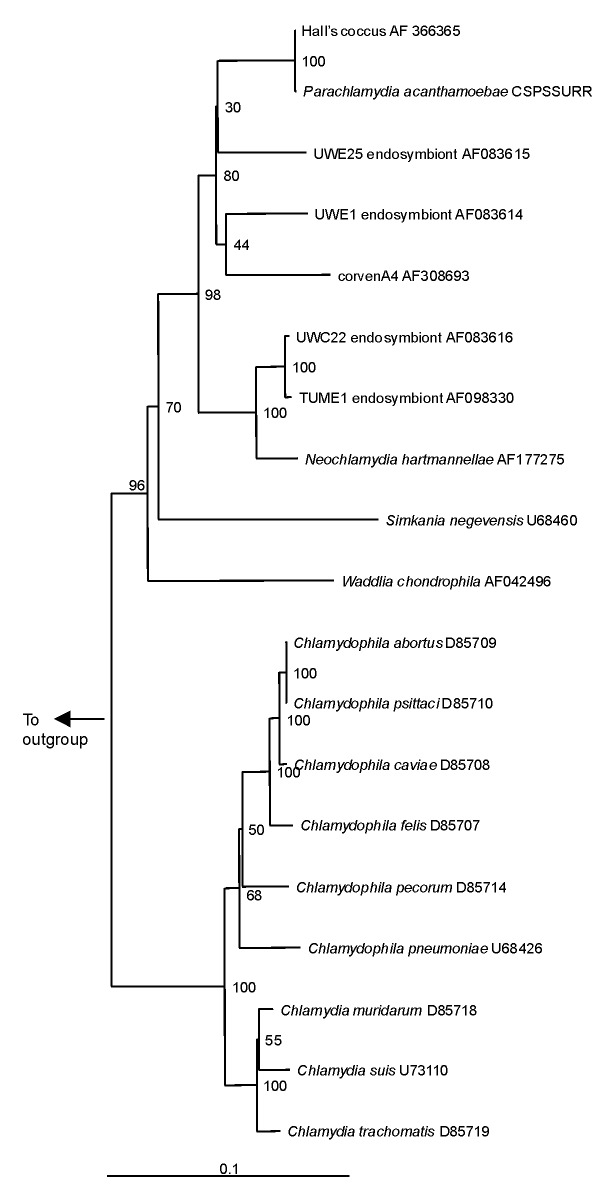
Neighbor-joining phylogenetic tree of the 16s rRNA gene sequence of *Chlamydiales*, including *Chlamydiaceae, Parachlamydiaceae,* and *Simkaniaceae,* compared with *Legionella pneumophila* (M 59157) as outgroup. Bar represents estimated evolutionary distance. The numbers at each node are the results of bootstrap analysis; each value is derived from 100 samples.

These *Chlamydia*-like endosymbionts are small Gimenez-stained (*43*) coccoid bacteria (Figure 1) that naturally infect amoebae and are inconsistently stained with Gram stain. Electron micrographs of *Acanthamoeba* demonstrate the presence of bacteria at different developmental stages typical of the *Chlamydiales,* such as elementary and reticulate bodies (Figure 2). A new *Parachlamydiaceae* family was proposed (*44*) that forms a sister taxon to the *Chlamydiaceae*, as it has a *Chlamydia*-like cycle of replication and 80% to 90% homology of ribosomal RNA genes. This family comprises two genera, of which the type strains are *Parachlamydia acanthamoebae* (*17*) and *Neochlamydia hartmanellae* (*45*). Members of the *Parachlamydia* were proposed to have at least 95% homology of the 16S or 23S rRNA genes with *P. acanthamoebae* (*44*)*.* However, comparison of the 16S rRNA gene sequences of four additional *Parachlamydia* with *P. acanthamoebae* showed substantial phylogenetic diversity within this genus (Figure 3), with 91.2% to 93.1% 16S rRNA gene sequence homology with *P. acanthamoebae* (*46*). The ecologic loci and prevalence of the *Parachlamydiaceae* are unknown, but the latter could be underestimated, as this fastidious gram-negative bacteria was recovered only by amoebal cocultures, a procedure not performed routinely on clinical samples. Moreover, these *Chlamydia*-like organisms have potential for widespread dissemination, as they are mostly endosymbionts of *Acanthamoeba*, a free-living amoeba with worldwide distribution (*27*).

## Strains of *Parachlamydiaceae*

Nine strains of *Parachlamydia* have been described (Table). The first, *P. acanthamoebae,* was identified within *Acanthamoeba* BN9, an amoeba recovered from the nasal mucosa of a female volunteer (*17*). Its 16S rRNA sequence had 88.2% homology with *Simkania negevensis* and 87% homology with *Chlamydophila pneumoniae* (*17*). The second, Berg17 endosymbiont, also isolated from the nasal mucosa of a female volunteer, seems to have an rRNA signature similar to that of the Bn9 endosymbiont, as demonstrated by the binding of the Bn9_658_ hybridization probe designed for in situ identification of *P. acanthamoebae* (*17*). The third, Hall’s coccus, was found in an *Acanthamoeba* isolated from water taken from a humidifier in a case of humidifier-associated fever in Vermont (*16*). Its 16S rRNA gene sequence had >99% similarity with that of Bn9 endosymbiont and 86% to 87% with those of the four recognized *Chlamydia* species (*16*). Two additional *Parachlamydiaceae,* UWE1 and UWE25, were also found to infect *Acanthamoeba.* Both amoeba strains were recovered from soil samples from Washington State (*46*). A sixth strain, UWC22 endosymbiont, infected an *Acanthamoeba* recovered from infected corneal tissues (*46*). TUME1 endosymbiont was found in an amoeba recovered from municipal sewage sludge in Germany (*46*). The eighth strain, *Neochlamydia hartmannellae*, is the only strain of *Parachlamydiaceae* isolated from *Hartmanella vermiformis.* It did not grow on *Acanthamoeba* sp. or *Naegleria*, and its 16S rRNA gene sequence had only 92% homology with that of *P. acanthamoeba* and varied from 91.6% to 97.1% with the four latter endosymbionts of *Acanthamoeba* (*45*). The last one, CorvenA4, could not be isolated. Only its 16S rRNA sequence was retrieved from a respiratory sample (*47*).

**Table:** Strains of *Parachlamydiaceae*

Strain	Sample, context and location	Host^a^	% 16S rRNA homology^b^		Ref
			to BN9	to *C. pneumoniae*^c^	
BN9 endosymbiont	Nasal swab of female volunteer, Germany	*Acanthamoeba* sp. strain BN9	100	87.6	17
Berg17 endosymbiont	Nasal swab of female volunteer, Germany	*Acanthamoeba maurianiensis*	na^d^	na^d^	17
Hall’s coccus	Water sample, humidifier fever, Vermont	*Acanthamoeba* sp.	99.6	87.4	16
UWE1 endosymbiont	Soil samples, Washington State	*Acanthamoeba* sp. strainUWE1	93.7	86.6	46
UWE25 endosymbiont	Soil samples, Washington State	*Acanthamoeba* sp. strain UWE25	93.2	86.8	46
UWC22 endosymbiont	Infected corneal tissues, Washington State	*Acanthamoeba* sp. strain UWC22	91.3	87.3	46
TUME1 endosymbiont	Municipal sewage sludge, Germany	*Acanthamoeba* sp. strain TUME1	91.0	87.2	46
*Neochlamydia hartmannellae*	Water system of a dental unit, Germany	*Hartmanella vermiformis*	91.5	86.8	45
CorvenA4	Bronchoalveolar washing, France	na^e^	91.4	85.0	47

^a^Bacterial strains were identified in free-living amoebae, isolated by culture on nonnutrient agar. ^b^Estimated with Clustal W ^63^ available on the website of Pôle Bio-Informatique Lyonnais, Lyon, France (http://pbil.ibcp.fr/). ^c^16S rRNA of *Chlamydophila pneumoniae* strain N16 (GenBank accession number U68426). ^d^Berg17 endosymbiont was shown to have a similar rRNA signature from Bn9 endosymbiont (binding of the Bn9_658_ hybridization probe designed for in situ identification of *Parachlamydia acanthamoebae*); however, the 16S rRNA sequence of that strain is not available. ^e^Direct polymerase chain reaction amplification and sequencing from DNA extracted from the respiratory sample; no strain was isolated.

## Pathogenicity

### Rationale for Potential Pathogenicity

Intra-amoebal growth may increase the virulence of some intracellular bacteria (*39*), prompting concern that other intracellular bacteria recovered from amoeba, such as the *Parachlamydiaceae,* could be pathogenic. Indeed, a bacterium able to survive exposure to the lytic enzymes of amoebal phagolysosomes would probably also survive the lytic activity of macrophages. This hypothesis is supported by the fact that mutants of *Legionella* that have similar cytotoxic defects and intracellular replication in mammalian macrophages and protozoa have been isolated (*48*), suggesting a common adaptive mechanism to the intracellular environment. Moreover, *Parachlamydia* can adapt to mammalian cells, as demonstrated by successful passage from an amoebal host to Vero cells (a monkey cell line) (*17*). Additional arguments in favor of a pathogenic role of the *Parachlamydiaceae* are that *Chlamydia pneumoniae*, a well-recognized agent of pneumonia, was shown to infect free-living amoebae (*19*) and that another member of the *Chlamydiales*, *Simkania negevensis* (*49*,*50*), which has 88% homology with *P. acanthamoebae* (*46*), has been shown to cause pneumonia in adults and acute bronchiolitis in infants (*51*,*52*).

Strong evidence that some *Parachlamydiaceae* could be pathogenic came from the identification of Hall’s coccus in an amoeba isolated from the source of an outbreak of humidifier-associated fever in the United States, as well as related serologic studies (*16*). In a study of 500 patients with pneumonia, fourfold rising titers against Hall’s coccus were observed in two patients and convalescent-phase antibodies in three others (*53*). In a second study, two patients had convalescent-phase antibodies (*16*). These results were recently confirmed: 8 (2.2%) and 3 (0.8%) of 371 patients with community-acquired pneumonia were seropositive (titer >1/50) or had a fourfold rise in *Parachlamydia* antibody titers compared with none of 511 healthy study participants (*54*). The recent identification of a 16S rRNA gene sequence of *Parachlamydiaceae* from bronchoalveolar lavage provides additional evidence of potential pathogenicity (*47*). However, the contamination of this specimen by an amoeba harboring the *CorvenA4-Parachlamydia* could not totally be ruled out. These findings should be interpreted cautiously as water contamination probably led to the initial false attribution of *Afipia felis* as the causative organism of cat-scratch disease (*55*). The identification in respiratory tract specimens of three new *Chlamydia*-like strains, which had phylogeny closer to that of the *Parachlamydiaceae* and *Simkaniaceae* than the *Chlamydia* and *Chlamydophila* (*56*), is an additional argument in favor of a role of the *Parachlamydiaceae* in the pathogenesis of respiratory diseases.

In addition, a patient with adult Kawasaki syndrome was found to have a fourfold rise in antibody titer to *P. acanthamoebae* (*54*). A possible relationship between a previous respiratory infection and Kawasaki syndrome has already been reported (*57*,*58*). Thus, the role of *Parachlamydia* in the pathogenesis of Kawasaki syndrome should be explored further.

As *Parachlamydia* could potentially be resistant to lytic macrophages enzymes for years, it could enhance chronic inflammatory disease or chronic pathogenic mechanisms, such as the one leading to vascular damage. A role of *Parachlamydiaceae* in the pathogenesis of arteriosclerosis is suggested by the presence in an abdominal aneurysm specimen of a *Chlamydia*-like strain that had a sequence closer to that of *P. acanthamoebae* than to *Chlamydia*, *Chlamydophila,* and *Simkaniaceae* (*56*). Some serologic studies have suggested that *Chlamydophila pneumoniae* could play a role in the pathogenesis of arteriosclerosis (*59*,*60*), although this observation was not confirmed in other studies (*61*,*62*). Such a discrepancy might result from serologic cross-reactions or confounding by a pathogen such as *Parachlamydia,* which in light of its homology could share epitopes, mode of transmission, or both with *C. pneumoniae*.

Based on this rationale, one may hypothesize that some *Parachlamydiaceae* could cause pneumonia. Thus, patients with nosocomial or community-acquired pneumonia of unknown etiology should ideally receive an extensive diagnostic work-up, including testing for *Parachlamydia*. In addition, patients with arteriosclerosis and Kawasaki disease or other infectious syndromes of unknown etiology should perhaps be tested for *Parachlamydia*. As *Parachlamydia* strains were all identified within free-living amoebae, recent history of swimming in ponds, rivers, or swimming pools might prompt a specific diagnostic approach.

### Diagnostic Methods

No diagnostic tool is commercially available. Because of the fastidious nature of *Parachlamydiaceae*, molecular biology is probably the easiest and cheapest diagnostic approach. Serologic testing is also promising; however, it requires antigen and a laboratory capable of performing amoebal coculture. Serologic results may be useful for epidemiologic studies, as they may provide information on past or present contact with the antigen. Both molecular and serologic methods may yield results in <24 hours.

Although time-consuming, culture-based diagnostic methods have the advantage of enabling the recovery of strains. These methods encompass two main approaches. The first one directly targets the recovery of *Parachlamydiaceae,* with amoebae used as cell background. A convenient broth for amoebal coculture is Page's modified Neff's amoeba saline (PAS) (*10*), which is preferable to Nelson's and peptone-yeast extract-glucose medium because PAS is devoid of nutrients, thus reducing overgrowth of potential contaminants in clinical samples. Although incubation at 37°C may be ideal for bacterial recovery, lower temperatures (30°C–35°C) are generally used to prevent amoebal death or encystment (*12*,*13*,*20*). The coculture should be examined regularly for amoebal lysis or Gimenez-positive cocci. The second culture-based method is designed to recover free-living amoebae, which will then be examined for the presence of endocytobionts. Briefly, amoebal culture is performed by adding the clinical sample to nonnutrient agar (1.5 g agar in 100 mL PAS) supplemented with living *Enterobacter cloacae* or *Escherichia coli*, incubating at 25°C–30°C, and examining the plate daily for the presence of amoebae. To date, all *Parachlamydiaceae* strains have been recovered by the second approach.

## Future Directions

The role of *Parachlamydia* sp. as an emerging pathogen needs to be confirmed. In view of the genetic diversity of the *Parachlamydiaceae* (*46*), their phylogeny needs to be elucidated, as the various species could be associated with species-specific pathogenicity. Search for additional *Parachlamydia* strains in hospital water systems could help define potential nosocomial exposures. Because the *Parachlamydiaceae* are difficult to culture, simpler approaches are being developed, including serologic and molecular tests. These methods could be performed on a large number of samples from both healthy and ill persons. Patients with community-acquired pneumonia, nosocomial pneumonia, Kawasaki disease, and arteriosclerosis should be tested. Increased resistance to antimicrobial drugs, which may be associated with intra-amoebal growth, is another promising area for future study.
